# Industrial Legacy and Glassmaking: Ecological and Human Health Risk Assessment in Paraćin, Serbia

**DOI:** 10.3390/toxics14040320

**Published:** 2026-04-12

**Authors:** Predrag Miljković, Jelena Beloica, Snežana Belanović Simić, Stefan Miletić

**Affiliations:** Department of Ecological Engineering for Soil and Water Resources Protection, University of Belgrade—Faculty of Forestry, 11030 Belgrade, Serbia; jelena.beloica@sfb.bg.ac.rs (J.B.); snezana.belanovic@sfb.bg.ac.rs (S.B.S.); stefan.miletic@sfb.bg.ac.rs (S.M.)

**Keywords:** historical pollution, PTEs, PAHs, pollution indices, carcinogenic risk, health indices

## Abstract

The glass industry contributes to long-term soil contamination. This study assesses the impact of over 150 years of industrial activity and over a century of glassmaking processes in the Serbian Glass Factory in Paraćin. Focusing on potentially toxic elements (PTEs) and polycyclic aromatic hydrocarbons (PAHs), ecological and human health risks were evaluated. Sampling was conducted at 34 locations within the factory area, including 33 soil samples (0–30 cm) and one industrial waste (IW) sample. Soil analyses indicate that Zn, Ni, Cu, and Cd exceeded both the maximum permissible concentrations (MPCs) and remediation values (RVs) in many samples, while As and Hg showed fewer exceedances. Based on the Potential Ecological Risk Index (RI), about 33% of soil samples were moderately to highly polluted, and Cd, Pb, As, and Hg were identified as the main contributors. High levels of PAHs and PTEs reflect the cumulative impact of long-term industrial operations, a historical fire, and secondary sources, including traffic-related emissions from nearby highways. These factors resulted in elevated total carcinogenic risk (TCR) for Ni, Cr, and As. This study highlights soil contamination and associated health risks at the glass factory, emphasizing the need for environmental monitoring and management.

## 1. Introduction

Soil pollution is one of the most challenging issues and refers to the presence of harmful chemicals or elements in soil, higher than what is considered a “normal concentration” [1,2]. It can affect biological and physicochemical soil characteristics [3,4]. Besides naturally caused accidents (floods, wildfires, earthquakes, etc.) [5,6], the most common cause of pollution comes from human activities: traffic, agriculture, mining, wastewater, landfills, various industries, etc. [2,7]. Among these, the glass industry, although often overlooked, also contributes to environmental pollution through its diverse facilities, raw materials, and production processes [8,9,10].

Glassmaking techniques have changed little since the Middle Ages, while most of the changes are attributed to the additives used for various types of glass or combustion fuels needed for the melting process [11]. The main raw materials are silica sand, soda ash, limestone, feldspar, recycled glass, etc. [11,12]. Minor ingredients include various sulfates and potentially toxic elements (PTEs) that may be used to produce colored and coated glass. PTE residues can be locally present in significant concentrations [11]. Emissions to air (over 80%) come from the melting furnaces and include combustion gases and particles from the furnace, e.g., dust, NO_x_, SO_x_, chlorides, fluorides, etc. PTEs and other particles and pollutants, present as impurities and generated during raw material processing [13], are released and deposited in wastewater and soil around facilities. In the glass production process, wastewater is discharged, with potential pollutants originating from coating material, toxic compounds, or organic compounds from lubricants [14]. These lubricants are almost exclusively based on mineral oils derived from the distillation of crude oil, and they most often contain PAHs [15,16].

Glass production in 2024 is estimated to be nearly 830 billion bottles and containers, while associated carbon dioxide emissions were approximately 95 million metric tons in 2022 [17]. Over 160 plants in EU countries have a production of over 20 million tons of glass per year [18], while in Serbia, 22,919 tons of hollow glass were produced in 2023, with a Serbian glass factory being the only significant domestic producer of packaging glass. Today’s factory facilities were built on the site of a broadcloth factory, destroyed in a fire in 1904, reflecting a complex industrial legacy. In addition, other potential sources of pollution may be considered in terms of how much they contribute to the overall contamination. For example, some studies have documented the impact of traffic volume of the roads and highways on soil contamination [19,20,21,22].

Some studies have shown elevated concentrations of PTEs and their mobile forms in soils near glassworks or glass landfills [23], while multi-decadal glass production has a negative impact on the ecosystem [24] and human health [25]. Some PTEs (lead, arsenic, nickel, chromium, cadmium, antimony, etc.) are used as pigments and agents in the glass industry for different purposes, such as purification, (de)colorization, removing bubbles from the glass during melting, increasing shine and refraction in crystal glass, etc. [14,23,26,27,28,29]. Despite the known environmental issues (air, water and soil contamination) primarily through emissions, waste, and historic operational practices, soil pollution in the glass industry [8,9,10] remains insufficiently addressed and reported in the scientific literature. In that regard, this research contributes to addressing this gap by assessing soil pollution and health risk due to various pollutants and potential sources related to the glass industry. The probability of adverse effects due to one or more pollution sources is considerable, and it can pose a risk to the environment and human health [7,30,31,32].

The aim of this study is to determine the impact of glass production on the current state of the soil. Besides basic physical and chemical soil characteristics, special attention is given to pollutants such as PTEs and polycyclic aromatic hydrocarbons (PAHs). Although some of the pollutants may be directly related to the production of glass, some may be the result of historical pollution or other nearby anthropogenic activities. Thus, this study aims to identify critical pollutants and their interaction under given conditions. Environmental and human health indices were used to evaluate the degree of soil contamination and potential health risk to workers in the factory facilities. The findings will provide the basis for measures and technologies that will reduce pollution and its negative impact on the environment, integrating the legacy of historical activities of this industrial emblem of Serbia.

## 2. Materials and Methods

### 2.1. Serbian Glass Factory

**Figure 1:**
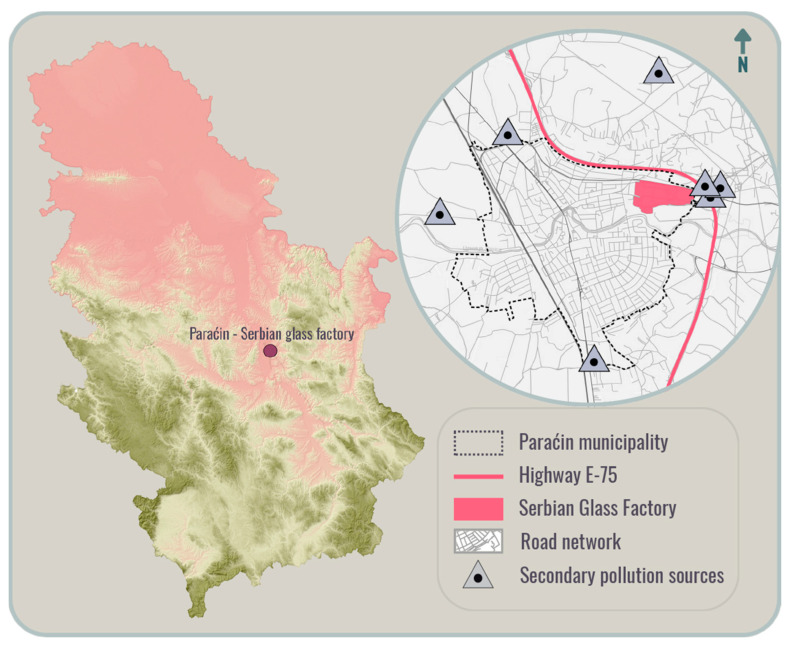
Study area—glass factory in Paraćin.

The Serbian Glass Factory (now Vaider Srpska fabrika stakla) is located in Paraćin, an industrial city in Central Serbia (Figure 1). Paraćin is situated at an elevation of 130 m above sea level, along the banks of the Crnica River, and about 4 km from the Great Morava River. The municipality covers a large part of the fertile Pomoravlje region. The industry began to develop in the early 19th century, and Paraćin became one of the most significant economic centers in Serbia at that time. Also, due to its proximity to major transportation routes (railway and first-class state highway A1/E75), with access to markets in Serbia and the Balkans, this city is of strategic importance. Among other industries spread throughout the city, the glass factory complex in Paraćin covers an area of 26.1 ha, where glass production, research, and administrative buildings are located.

### 2.2. Historical Background

**Figure 2:**
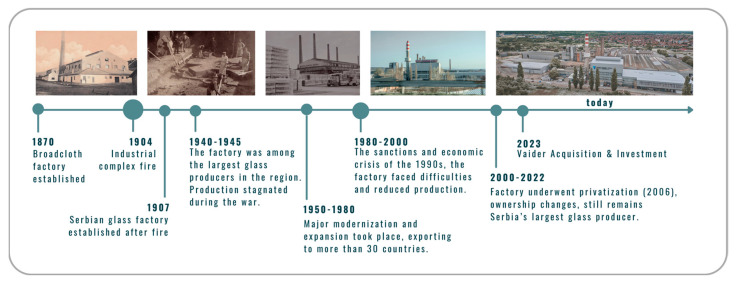
Historical industrial overview of the studied area. Photo sources [33,34,35,36].

The recorded industrial activity (Figure 2) in the studied area dates back to 1870, when the Minh brothers established a broadcloth factory, which was partially burned down in 1904.

What was preserved from the factory infrastructure after the fire, especially the steam plant, facilitated the Serbian Glass Factory construction in 1907 and the start of glass production. The Serbian Glass Factory became the most important factory producing luxury hollow glass for catering and packaging for the wider region of the former Kingdom of Yugoslavia. It was a symbol of industrial and cultural heritage of Serbia and former Yugoslavia. For more than a century, the factory had a significant role in national and regional economic development. As a hub of social activity, with its own magazine, sports club, cultural sections, and publishing initiatives, the factory was deeply integrated into local life. In addition, it also contributed to artistic expression, everyday aesthetics, and the social and cultural life of the community [37]. Its products are now kept in ethnological collections of museums in Paraćin, Jagodina, and other cities as a permanent emblem of the importance of the glassmaking tradition in the cultural heritage of Serbia.

### 2.3. Soil Sampling

**Figure 3:**
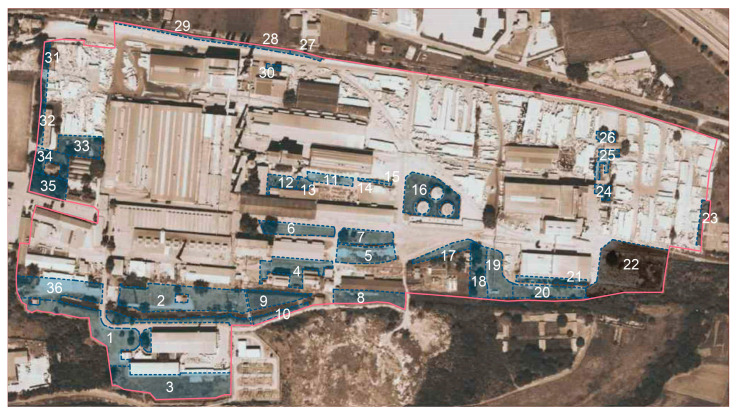
Study area (Serbian Glass Factory) outlined in pink, with soil sampling sites shown as blue shaded areas, except sites 22 and 23.

According to the pedological map of Serbia, the Serbian glass factory in Paraćin is situated on alluvial-meadow soil (Fluvisol). However, due to industrial development, starting from the broadcloth factory, and later the glass industry, the area within the factory site has most likely been gradually infilled with construction and industrial waste (as confirmed during soil sampling), which has transformed the natural into anthropogenic soil, classified as technogenic soil (Technosol) according to WRB classification [38]. Within the factory production complex, 36 sampling sites were selected for soil sampling (4.06 ha in total) (Figure 3), of which 35 were soil samples, and one sample was from the industrial waste disposal site. However, samples 22 and 23 were not taken into consideration for analysis due to improper sampling. Sampling areas are smaller spatial units in relation to buildings and traffic infrastructure.

Soil sampling was conducted within the 0–30 cm surface layer with an auger. Each composite sample consisted of three to six individual subsamples, depending on the area size and skeletal content. Where possible, a square sampling pattern was applied (from each corner and at the intersection of the diagonals). Other cases involved linear or zigzag sampling. About 1 kg of sample was packed in plastic bags, marked, and tightly closed.

### 2.4. Laboratory Analysis

The samples were air-dried in the laboratory and prepared for physicochemical analysis. All analyses were performed according to the standards in use in laboratory practice at the time of analysis. The soil particle size distribution was determined after treating the samples with sodium pyrophosphate. Soil fractionation was performed using a combining of the pipette method and the sieve elution method. Soil textural classes were determined according to SRPS ISO 11259:2005 [39]. Soil reaction (pH) was determined by an electrometric method, using a standard laboratory pH meter (SRPS ISO 10390:2007 in accordance with ISO 3696:1987) [40,41]. CaCO_3_ was determined by Scheibler calcimeter–volumetric determination (standard laboratory apparatus) (SRPS ISO 10693:2005) [42]. Organic carbon was determined by SRPS ISO 13878:2005 [43] and SRPS ISO 10694:2005 [44], respectively. The fluoride content was determined using fusion with NaOH followed by measurement using an ion-selective electrode. Cl^−^ was determined by ASTM D512-19 [45], Method B. PTEs were determined after aqua regia digestion. Analyses were conducted in two accredited laboratories. The contents of Zn, Cu, Pb, Cr, Cd, Ni, and Fe were measured using atomic absorption spectrometry (AAS) (Thermo Fisher Scientific, Waltham, MA, USA). The concentrations of As, Sb, and Se were determined by inductively coupled plasma optical emission spectrometry (ICP-OES) (Thermo Fisher Scientific, Waltham, MA, USA) (ISO 11466:1995) [46]. The Hg content was determined separately using a direct mercury analyzer (Milestone, Bergamo, Italy) method and atomic absorption spectrometry. Polycyclic aromatic hydrocarbons (PAHs) in soil samples were determined in accordance with ISO 18287:2006 [47] by a gas chromatographic method with mass spectrometric detection (GC–MS) (Agilent Technologies, Santa Clara, CA, USA).

### 2.5. Pollution Index

The Pollution Index (PI) is used to determine which PTE poses the greatest threat to the environment. It is also used to determine certain complex pollution indices. It is calculated according to the formula [48]:

(1) PI=Cn/GB

where Cn—concentration of PTEs in soil; and GB—background concentration (values adopted from the project for the locality in the vicinity of the study area [49]).

The Potential Ecological Risk Index (RI) is an index used to assess the degree of ecological risk that may arise from elevated concentrations of PTEs in water, air, or soil. This index was introduced by Håkanson and is calculated according to the formula [48]:

(2) $$\mathrm{RI}=\sum_{i=1}^{n}E_{r}^{i}$$

where n—the total number of PTEs being analyzed; and Er—the individual ecological risk index calculated according to the formula:

(3) $$E_{r}^{i}=T_{r}^{i}\times \mathrm{PI}$$

where Tr is the toxic response factor of a particular PTE and PI is the calculated individual Pollution Index. The Tr values were taken from the literature and for the following PTEs: Zn—1, Cu—5, Pb—5, Ni—5, Cr—2, Cd—30, As—10, Hg—40, Sb—7 [50,51]. Five classes are defined for the individual ecological risk index (Er) as follows: Er < 40: low potential ecological risk; 40 ≤ Er < 80: moderate potential ecological risk; 80 ≤ Er < 160: considerable potential ecological risk; 160 ≤ Er < 320: high potential ecological risk; and Er ≥ 320: very high ecological risk.

The Potential Ecological Risk Index (RI) is defined in four classes as follows: RI < 150: low environmental risk; 150 ≤ RI < 300: moderate environmental risk; 300 ≤ RI < 600: considerable environmental risk; and RI ≥ 600: very high environmental risk.

### 2.6. Health Indices

According to USEPA [52], human health risk is assessed based on the possible hazardous effects of PTEs [30,53]. Both the non-carcinogenic health risk index (HI) and total carcinogenic risk index (TCR) were assessed using USEPA-specific parameters and screening values for the composite worker soil exposure scenario (Tables S1 and S2). The study included three types of exposure: ingestion, inhalation, and dermal contact. The average daily doses (ADD) (mg kg^−1^ d^−1^) were estimated using the formulas:

(4) $$ADDing=[(CS\times IngR\times EF\times ED)/(BW\times AT)]\times 10^{-6}$$

(5) ADDinh=(CS×InhR×EF×ED)/(PEF×BW×AT)

(6) $$ADDder=[(CS\times SA\times SL\times ABS\times EF\times ED)/(BW\times AT)]\times 10^{-6}$$

The hazard quotient (HQ) and hazard index (HI) were calculated as follows:

(7) HQ=ADD/RfD

(8) $$HI=\sum {HQ}_{i}$$

The estimate for carcinogenic risk (CR) for workers was calculated only for elements with available carcinogenic slope factors (Pb, Ni, Cr, Cd, As), based on the formula:

(9) CR=ADD×CSF

The total carcinogenic risk (TCR) for the analyzed PTEs is expressed as the sum of carcinogenic risks for different exposure types. Negative effects for the non-carcinogenic risk can be expected when HI values are greater than 1 [30,32,53], while carcinogenic risk is expected with TCR values above 10^−4^ and acceptable when in the range between 10^−6^ and 10^−4^ [30,32,53,54,55]. Details for the parameters used in the assessment of HI and TCR are given in the Supplementary Materials (Tables S1 and S2). It should be noted that total chromium was analyzed without speciation between Cr(III) and Cr(VI), and therefore the carcinogenic risk likely represents a conservative estimate. TCR was calculated according to USEPA, without including GIABS, due to lack of site-specific toxicological data and to preserve the standard slope factor formulation. CSF for ingestion, inhalation, and dermal exposures were adopted from previously published studies and based on international risk assessment frameworks (Table S1).

### 2.7. Statistical and Spatial Analysis

Descriptive statistical analysis as well as Pearson correlation coefficients were performed in Statgraphics Centurion (Version 18.1.12). Factor analysis (FA) was performed using standardized data to identify potential grouping patterns and common sources of PTEs. The suitability of the data for factor analysis was assessed using the Kaiser–Meyer–Olkin (KMO) measure and Bartlett’s test of sphericity. Factors with eigenvalues > 1 were retained, and Varimax rotation was applied to improve the interpretability [56]. Boxplots and the pollution risk matrix were generated in Origin 2019b (OriginLab Corporation, Northampton, MA, USA). Spatial analysis and maps were generated in QGIS (v.3.16.4). Georeferenced satellite imagery obtained from the GeoSrbija portal (https://a3.geosrbija.rs/) (accessed on 8 April 2026) was used as a background layer, cropped to the study area, and used for illustrative purposes within AutoCAD 2017 (Autodesk Inc., San Rafael, CA, USA).

## 3. Results

### 3.1. Physicochemical Properties

For the analyzed physicochemical properties, basic statistical parameters are given in Table 1. According to the mechanical composition, the intermediate classes dominate, which are: clay loam (27%) and sandy clay loam (24.5%). This implies moderate water retention and nutrient capacity. The presence of sandy loam and loamy sand (36.5%) indicates improved drainage and aeration. However, since soil samples were taken from the glass factory facility site, the texture has likely been altered by anthropogenic factors. Soil compaction, industrial byproducts, or soil mixing during past site activities (textile industry and former glass factory) led to changes in the soil, which in turn resulted in historical pollution. Sand and clay were present in 6% and 3% of samples, respectively.

The pH values range from 7.28 to 8.62 (Table 1), indicating alkaline soil conditions. Also, approximately 80% of soil samples are moderately to strongly calcareous, which may additionally influence PTEs. Organic carbon ranged between 10.71 and 164.48 mg kg^−1^ and plays an important role in oxidizing and reducing PTEs in soil [57], affecting their bioavailability and leaching into water bodies.

As a fining agent in the glassmaking industry, fluorides and chlorides can be found as byproducts in air, water, and soil due to various processes, from melting to glass fining. Hydrofluoric acid is used for etching glass [58], after which excess chemicals end up as a waste. In the study area, the fluorides exceeded MPC in two samples, while the average concentration is 292.23 mg kg^−1^. On the other hand, chlorine is associated primarily with alkalis used in soda glass manufacture [59], which from the time of Mesopotamia and Roman Empire up to the 19th century had a relatively high concentration of chlorine in soda–potash–lime–silica glass [60]. Although Cl^−^ in the studied soil is in normal concentrations, the same authors stated that chlorine is volatile during glass manufacture (melting) and that similar problems arise with other elements, such as arsenic, zinc, and selenium, which in turn accumulate in soil through atmospheric deposition or waste disposal. Glass production over many decades may thus have a negative impact on soil and flora, altering soil chemistry and plant health [24].

### 3.2. Potentially Toxic Elements and Regulatory Values

Regarding the measured concentrations of PTEs in the study area, it can be concluded that the soil shows clear signs of contamination, based on the number of samples exceeding maximum permissible concentrations (MPCs) and remediation values (RVs), provided in the regulation of the Republic of Serbia [61] (Table 2). The PTEs that most frequently exceeded MPC in soil expressed as the percentage of samples are as follows: Ni (91.2%), Cd (51.6%), Pb (32.4%), Zn (26.5%), Cu (26.5%), and As (8.8%). Although Hg and Sb were detected in only four and five samples, respectively, their concentration exceeded the MPC in three and one of them, respectively. According to authors [20,24,30,31,32,50,62,63,64], high PTE concentrations in soils are detected in the areas with industrial activities, mines, tailings, traffic, agriculture, areas affected by floods, etc. The overview of PTEs is shown in Table 2.

### 3.3. Polycyclic Aromatic Hydrocarbons

Out of 34 soil samples, PAHs were not detected in only six of them. However, low-molecular weight (LMW) PAHs, which exhibit high volatility, were more commonly found in the soil, likely derived from the continuous emission source, atmospheric deposition, or recent low-temperature combustion sources (traffic, solid fuel combustion, etc.). Apart from anthracene, which was detected in four samples, naphthalene and phenanthrene were detected in 21 and 19 samples, respectively (Table 3). PAHs were not detected in the IW sample.

**Figure 4:**
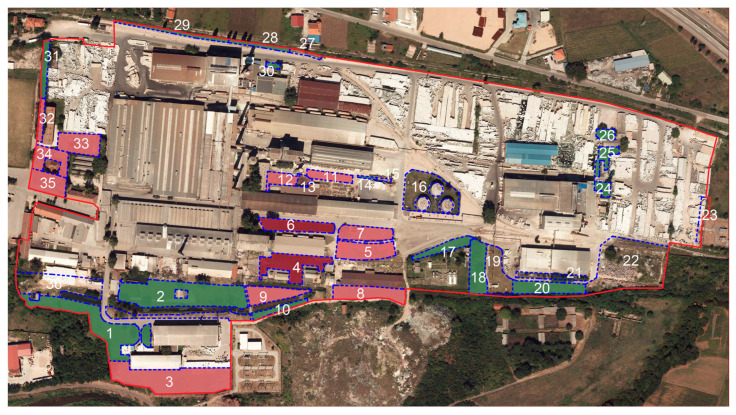
Locations with no detected PAHs (unshaded), locations dominated by LMW PAHs (green), locations dominated by HMW PAHs (light red), and locations where total PAH concentrations exceeded the MPC (dark red).

On the other hand, high-molecular weight (HMW) PAHs were detected in a smaller number of samples, on average in 9.4 samples. Their concentration in most samples is below the limit of detection (0.03 mg kg^−1^). This may indicate either a low level of contamination or PAHs degradation over time. In samples where most HMW PAHs were detected, they contributed more than 78% of the total PAHs content. At sites where total PAH concentrations are above MPC according to regulation [61], HMW PAHs were particularly dominant, accounting for more than 80% of the total PAH mass. These sites are in the immediate vicinity of the main facility (Figure 4–dark red shading), where the waste material or combustion byproducts were probably disposed of. These sites may be considered priority zones for remediation, also considering the presence of elevated concentrations of Pb, Cd, and As.

### 3.4. Pollution Indices

**Figure 5:**
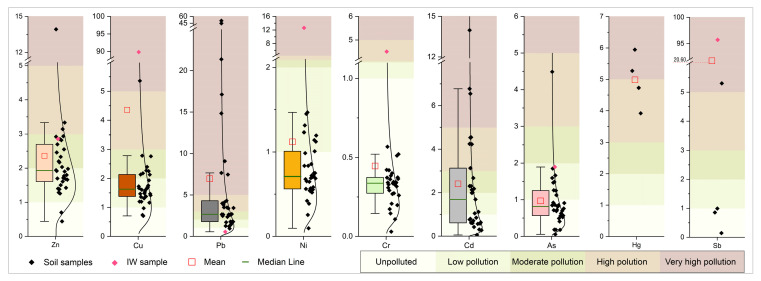
Boxplot representation of Pollution Index (PI) with contamination classes.

The individual Pollution Index indicates potential contamination by a particular PTE, and it is used in the calculation of complex pollution indices. In the studied area, the average values of the Pollution Index (PI) increase in the following order: Cr < As < Ni < Cd < Zn < Cu < Hg < Pb < Sb. The PI values range from 0.03 to 95.72. The largest range of values of this index is for Cu, Pb, and Sb. All soil samples for Cr fall into the class of unpolluted soils (except for the IW sample), whereas other PTEs (except Ni and Cr) have the lowest share in the unpolluted category. The most prominent PTEs with the greatest share in the categories of moderate to very high pollution were Zn (44%), Cd (41%), Pb (67.6%), and Hg (100%). Due to the low number of detected samples for Hg (*n* = 4) and Sb (*n* = 5), PI for these elements was visualized as scatter columns (Figure 5), as distribution-based visualization would not be meaningful. Boxplots for other PTEs include the lognormal distribution curve.

**Figure 6:**
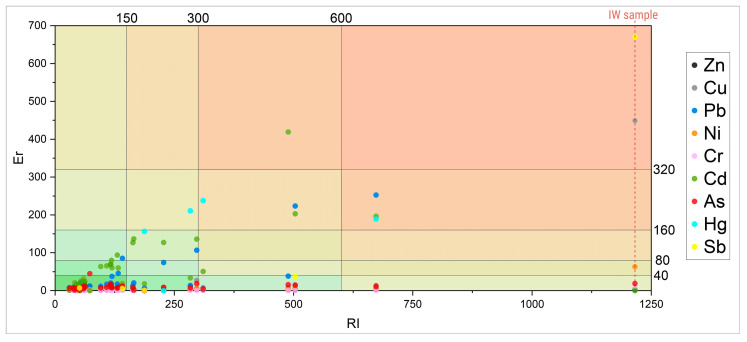
Contamination risk matrix based on individual (Er) and total (RI) ecological risk indices.

Analysis of the individual Er indices shows that Pb, Cd, and Hg (despite Hg being detected in only four samples) consistently exhibit the highest contributions to the total ecological risk (RI), suggesting that these elements play a key role in the overall contamination level. As shown in Figure 6, besides these elements, Sb, Ni, and Cu stand out as the elements with the highest Er values in the most contaminated sample (IW sample), thus contributing most significantly to the total RI index. Equally important, almost all soil samples are categorized as unpolluted with respect to As (based on the Er index); still, this element shows significant and steady contribution to the total RI.

### 3.5. Health Indices Analysis

The analysis of HI shows that all the values are below one, which is the limit value, and although low-level risk cannot be completely excluded, the non-carcinogenic risk is not expected for any of the analyzed PTE (Table 4).

**Figure 7:**
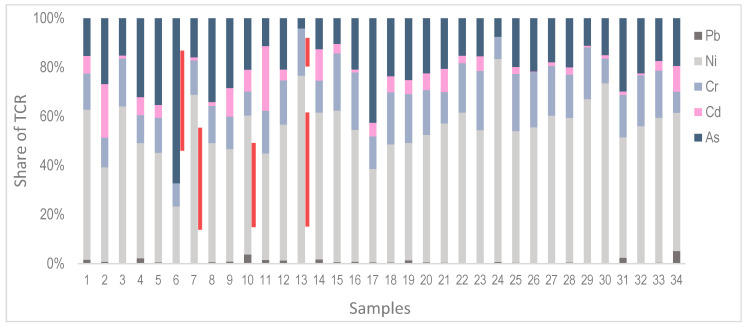
Relative contribution of individual PTE to TCR across samples. Red shading indicates unacceptable TCR values for the corresponding PTE.

However, if total carcinogenic risk (TCR) is analyzed, the risk is not calculated for Zn, Cu, Sb, and Hg, because carcinogenic slope factors are not available. The carcinogenic risk is regarded as negligible for mostly Pb (values are below 10^−6^). In most samples, TCR values are within the tolerable range (10^−6^–10^−4^) for Ni (91.2%), Cr (97.1%), Cd (82.4%), and As (97.1%). However, the total carcinogenic risk was estimated for Ni in three samples, and for Cr and As in one sample, for which the TCR is considered unacceptable (>10^−4^) (Figure 7). For all samples and PTEs, the dominant exposure type is through ingestion, while nickel consistently dominates in most samples in terms of its contribution to total TCR values.

### 3.6. Correlative Statistics

The analysis of soil samples reveals that besides soil fractions that do not correlate significantly with other soil properties or pollutants, there is significant variability among other soil properties and pollutants. Very weak or no correlation between soil fractions and most PTEs (Table S3) suggests that sorption may not be the dominant process. This may be due to long-term anthropogenic disturbances, which in turn resulted in the loss of natural pedogenetic development. Although increasing the pH decreases the solubility of zinc in soils [65], this element is highly positively correlated with pH (r = 0.54 **) (Table S3). This indicates a higher content of adsorbed or fixed Zn and not necessarily the soluble form. Besides soil reaction, Zn also exhibits significant correlation with CaCO_3_ (r = 0.77 ***) (Table S3). Calcium carbonate showed notable correlation with naphthalene (r = 0.47 *). Organic carbon (OC) exhibited positive correlation with Fe and As, strong correlation with phenanthrene and total PAHs, and negative weak correlation with soil acidity (Table S3). This finding is reasonable as it reflects the influence of industrial activity.

In the soil systems, arsenic is influenced by soil properties, such as pH, redox processes, organic matter, Fe-(oxyhydr)oxides, etc. [66], which is in accordance with our findings, i.e., strong correlation of As with organic carbon (r = 0.58 ***), Fe (r = 0.75 ***), and pH (r = −0.64 ***). In addition to this, As is correlated with total PAHs and Cd (Table S3). Moderate to high correlations are present among Cu, Ni, and Cr, as well as between Pb and Cd, indicating similar source or behavior mechanism in soil.

Although PAHs were not detected in all samples, the number of available combinations among individual PAHs with other parameters was sufficient to provide informative correlation (Table S3). The correlation did not include anthracene, indeno[1,2,3-cd]pyrene, and benzo(ghi)perylene due to a very small number of samples. Total PAHs content shows a very high correlation with individual HMW PAHs, moderate correlation for phenanthrene, and none for naphthalene (as the most frequently detected LMW PAH). It can be assumed that naphthalene contributes independently to total PAHs. Among analyzed samples, where both LMW and HMW PAHs were detected, naphthalene contributed on average 4.5% to the total PAHs concentration. Nevertheless, the naphthalene, the most detected PAH (21 soil samples), showed weak positive correlation with sand (r = 0.35) and weak negative correlation with both silt and clay (r = −0.34 each), none of which were statistically significant. The most significant correlations in the context of glassmaking processes are as follows: naphthalene with Zn (r = 0.46 *), Cr (r = −0.55 **), and Cl^−^ (r = 0.53 *); and phenanthrene with OC (r = 0.51 *), Cu (r = 0.51 *), Pb (r = 0.73 ***), and Cd (r = 0.65 **), while total PAHs are highly correlated with Fe (r = 0.68 ***), As (r = 0.79 ***), OC (r = 0.70 ***), and pH (r = −0.59 **). Other significant correlations were observed between most HMW PAHs and Fe or As (r > 0.8), calculated only for samples in which both the HMW PAHs and corresponding PTEs were detected (Table S3).

To further explore the relationships among PTEs and identify broader grouping patterns, FA was performed. The suitability of the dataset for FA was confirmed by the Kaiser–Meyer–Olkin (KMO) measure (0.657) and Bartlett’s test of sphericity (*p* < 0.05). Two factors with eigenvalues > 1 explained 84.3% of the total variance (Table 5).

Factor 1 is characterized by high loadings of Pb, Cu, Cd, and As and with a moderate contribution from Zn. These results potentially suggest a predominantly anthropogenic influence. In contrast, Factor 2 is dominated by Cr, Fe, and Ni, which indicate a geogenic origin. These results are consistent with the correlation analysis. However, FA provides a more integrated view of the relationships among elements.

## 4. Discussion

### 4.1. Factors Controlling PTEs and PAHs

Based on the correlation matrix (Table S3), three clearly expressed local groups of PTEs can be distinguished.

Group A (Ni-Cr-Cu) may be partly from the same geogenic origin or geomorphological influence. Although the study area is not directly associated with serpentinite soils, elevated Ni concentration may be indirectly related to this geological substrate, transported from surrounding areas rich in Ni and Cr. They originate from the mountains that influenced the geochemical properties of soils in alluvial plains on which the study area is located [49]. The average Ni concentrations in a large part of Serbia is 127.88 mg kg^−1^ [49], which exceeds the MPC level, and 58 mg kg^−1^ in Central Serbia [62]. The same authors stated that the Cr concentrations are 105.87 mg kg^−1^ and 48 mg kg^−1^, respectively. Although high Ni content in soil does not imply high Cr content, nevertheless, their strong correlation (r = 0.99 ***) suggests the same origin but a different transport and deposition mechanism over time. On the other hand, Cu is not a commonly associated element with Ni and Cr, as in serpentine rock. Nevertheless, their strong correlation, combined with the exceedance of the RV in IW sample, suggests an additional anthropogenic influence, i.e., industrial contamination. When the coloration of glass is desired, the oxides of Ni, Cr, and Cu are used [25], which may be the cause of elevated concentrations of these elements, and they persist over time. Before the glassmaking, there was a textile industry, especially for broadcloth production. According to the literature [67], from the second half of the 19th century, there was a shift from natural mordants (alum, potash, calcium salts, sodium chloride, vinegar, tartaric acid, and citric acid) to the ones where metal salts were used (specifically Al, Cr, Cu, Fe), which could also contribute to environmental pollution.

Group B (As-Fe) is represented by their strong correlation (Table S3) and potential As sorption to Fe oxides in the soil alkalinity conditions [68,69,70]. Although arsenic is mostly found naturally in soils near antimony mines and mineral deposits [32,49], its origin in the study area may be partly geogenic, as indicated by strong correlation with Fe, and partly from glassmaking activities, as a glass purifier or by burning arsenic-containing coal [26,28,71]. Under alkaline and dry conditions, arsenic dominates in the form of arsenate [68,69], which has a lower affinity for sorption on carbonate and clay (very weak or negligible correlation, (Table S3)) but can be retained in the presence of Fe oxides [70]. In Central Serbia, the average value of arsenic is 11 mg kg^−1^ [62]. In another study conducted in Central and Western Serbia [49], As concentrations are on average 12.98 mg kg^−1^. High As contents in Serbia are dominantly present in polluted environments [32,49,64] often exceeding limit and remediation values, not only in soil, but also in the air [63]. During most of the glass production history in the study area, there were no regulations on pollutants. Despite lack of historical data on pollutant emissions in this process, it is likely that the earlier melting furnaces emitted higher amounts of pollutants into the environment.

Group C (Cd-Pb) is characterized by more than 25% of soil samples in which both these PTEs exceeded their respective MPCs. Cadmium is often associated with lead ore deposits, as well as some limestone formations [49], and their correlation in the study area (r = 0.49 **) may be explained by the geochemical influence of upstream rock masses. On the other hand, both elements may also be associated with anthropogenic influence [31]. The glass factory in Paraćin used to make crystal glass, which according to the literature, is followed by the emission of gases and dust containing lead compounds [23]. Significant content of Pb and Cd may also be found at sites contaminated by glass waste [23,72], but also on decorated drinking glassware [73]. Moreover, both PTEs also correlate with phenanthrene, which may suggest a common source, such as the use of coal-fired furnaces and industrial combustion during the operational period of the glass factory. It is confirmed that high concentrations of As, Pb, Cd, and Zn are possible in the soils near glass factories, as well as elevated concentrations of mobile Al, As, Fe, and Pb [23].

PTEs’ mobility and availability are directly affected by pH [74,75]. Such levels of alkalinity (Table 1) has an impact on the retention mechanisms of some PTEs on solid surfaces [76], especially mobile and bioavailable forms. In addition to pH, carbonates also have an influence on Zn desorption and an increase in exchangeable Zn content [77].

Regarding PAHs, based on the literature [78,79], naphthalene adsorbs more on the clay fraction. This suggests its high volatility, mobility, or recent source of contamination, and therefore has not established its full sorptive potential. Considering the proximity of the E75 highway, as well as the fact that naphthalene and Zn are byproducts of exhaust gases, tire wear, asphalt abrasion, etc., and their moderate correlation (r = 0.46 *), it can be concluded that they partially come from the same source, distinct from that of the other PAHs. The observed correlation between phenanthrene and PTEs (Cu, Pb, and Cd) (Table S3) most likely reflects an uncommon origin (i.e., a shared source of these pollutants), but rather simultaneous processes, such as traffic in the vicinity of the factory and glass production. Additionally, OC correlates with total PAHs (r = 0.70 ***) as well as with most of the individual PAHs, indicating strong interaction in which the adsorption of organic pollutants is facilitated by the OC and not soil texture [80,81]. However, this mechanism does not apply equally to naphthalene due to its higher solubility and volatility. The correlation matrix (Table S3) may indicate different sorption mechanisms, interactions, and sources for the analyzed parameters.

To further support the observed relationships among PTEs, a factor analysis (FA) was applied to identify broader grouping patterns. Two dominant factors were distinguished, which explained 84.3% of the total variance (Table 5).

Factor 1 resulted in high loadings of Pb, Cu, Cd, and As and moderate contribution from Zn. These results indicate predominantly anthropogenic origin, which is associated with glassmaking activities. Other PTEs, such as Cr, Fe, and Ni, contributed to Factor 2, which is indicated by geological origin. While the correlation analysis highlights several smaller groups of elements, FA integrates these relationships into two broader factors, separating anthropogenic inputs from geogenic controls. This supports the interpretation of a complex contamination system influenced by multiple overlapping sources.

In contrast to PTEs, which were further evaluated using factor analysis, PAHs exhibited a more heterogeneous distribution and were therefore interpreted separately based on their occurrence patterns and correlations. One of the indicators of historical soil pollution is high OC and total PAHs content at certain locations (Figure 4, sampling points 4 and 6) near the main facility, where the total PAHs content exceeded MPC values. Significant positive correlations between As, Cu, and Cd with some individual PAHs (benzo[a] anthracene, benzo[k] fluoranthene, benzo[a] pyrene, chrysene, fluoranthene) indicate that these pollutants deposit together. Additionally, PAHs in soil near traffic and industrial areas were five times greater than those in suburban areas and are primarily associated with combustion [20]. A strong correlation between Fe and these PAHs further indicates that these pollutants are not only bound to OC but also to mineral fraction, particularly Fe oxides. OC is associated with Fe oxides through the formation of Fe–OC complexes, important in global soil carbon accumulation and stabilization [80]. Lower pH increases Fe solubility and thus enhances the formation of Fe-OC [82]. However, under alkaline conditions, Fe oxides are potentially stable and persist, reflecting long-term oxidation processes and industrial activity. In this context, organic carbon is partly of anthropogenic origin, associated with the combustion of fossil fuels, wood, waste, etc. Thus, the presence of PAHs may be used as a diagnostic indicator for anthropogenic origin of OC. In most samples, the LMW/HMW ratios were below one, indicating a predominantly pyrogenic origin, typical of industrial combustion processes.

### 4.2. Pollution and Health Indices

Analysis of the individual Er indices shows that Pb, Cd, and Hg consistently exhibit the highest contributions to the total ecological risk (RI), indicating that these elements play a key role in the overall contamination level. As shown in Figure 6, besides these elements, Sb, Ni, and Cu stand out as the elements with the highest Er values in the most contaminated sample (IW sample), thus contributing most significantly to the total RI index. Equally important, almost all soil samples are categorized as unpolluted with respect to As (based on the Er index); still, this element shows significant and steady contribution to the total RI. Regarding environmental pollution risk, as shown in Figure 6, a certain number of samples distinguish smaller clusters of simultaneously high values for both indices (Er and RI), predominantly driven by Pb and Cd. Additionally, although detected in only four samples, Hg also exhibits contribution in the same locations, which implies potential occurrence linked to similar deposition mechanisms or contamination pathways. These metals are persistent, toxic, and can bioaccumulate in the environment, entering the food chain. However, Cd is generally more mobile and soluble in soils. Moreover, contamination may be the result of industrial release of effluent and dust particles or volatilization and dispersion of these elements during coal combustion, while besides the topsoil, contamination with Cd may be found up to the depth of 2 m [83,84]. In a significant number of soil samples, As greatly contributed to elevated values of total risk index (RI), which can also be associated with atmospheric emissions from industrial complex [26,28,82]. Thus, As can be considered as an underlying component of the overall contamination.

Under current exposure scenarios, the HI values for observed PTEs reflect negligible non-carcinogenic risk. However, previous research suggests that for As, Pb, and Sb, risk is most affected by element concentration, followed by exposure [32]. In contrast, TCR values are generally negligible to tolerable but unacceptable for Ni, Cr, and As in sporadic samples, suggesting localized hotspots. High TCR values identified for these elements indicate heterogenous deposition. The discrepancy with ecological risk assessment, especially for the elements that show high pollution potential, indicates different perspectives and that low human health risk does not imply low environmental risk and vice versa. This is particularly important due to the processes and materials used in industry, while special attention should be given to the conditions and exposures inside facilities.

To place these findings in a broader context, measured concentrations can be interpreted in relation to international guidelines, such as the Canadian Soil Quality Guidelines (CCME), which define threshold values based on land use. Although a direct numerical comparison was not performed, the observed values indicate generally moderate contamination levels, with certain elements showing elevated concentrations that may suggest localized hotspots.

In addition, background values reported in the literature [49,85], which vary depending on soil type and geological conditions, are generally lower than the observed concentrations, indicating a predominantly anthropogenic origin of contamination. Similar observations have been reported in industrial areas, where elevated concentrations of PTEs are associated with long-term industrial activities and may pose potential environmental and human health concerns, emphasizing the need for continuous monitoring [86].

Identified contamination has important ecological and human health implications. Elevated PTE concentrations may degrade soil quality, affect microbial activity, and enable transfer into the food chain through plant uptake. The presence of carcinogenic elements indicates potential long-term health risks, particularly for workers. In addition, waste material and wastewater from the facility may affect surface and groundwater, degrading water quality and aquatic life. In this context, measures for environmental risk reduction are necessary, which may include detailed and long-term environmental monitoring and control of production areas and materials used in glassmaking. Regarding contaminated soil, remediation strategies should be implemented in accordance with national regulations. Such measures include removal of highly contaminated material and its safe disposal, as well as phytoremediation in case of mild contamination. Regarding human health risk, regular medical monitoring should be implemented, along with the appropriate protective measures in the working environment.

### 4.3. Industrial Legacy and Fire as Drivers for Soil Contamination

Besides glassmaking activities, fire can significantly affect physical and biogeochemical properties of soil and release and redeposit PTEs on the soil surface or into surface waters, which in turn can make it contaminated [87]. It takes years for soil to recover, while some studies show that some forest soils need several decades or more [88]. Release and deposition of PTEs may be the result of the combustion of vegetation and organic matter in soil, which could lead to the breakdown of organic–metal complexes but also due to the deposition of ash and charcoal. In such conditions, fire can release and remobilize PTEs from vegetation and soil organic matter in a series of complex processes [87]. According to [89], when multiple PTEs coexist in already contaminated soil, as is the case in the study area, competitive adsorption may occur.

Before the existence of regulations on pollutants, it can be assumed that all industrial activity, along with the fire accident in the study area, contributed to emissions. Although little is known of the textile factory, it most likely operated coal or wood-fired engines for textile processing. After more than two decades of operation, a fire that broke out in the textile factory could release various pollutants, including hydrophobic, poorly soluble, and persistent PAHs. Although there is no direct evidence that some of the PAHs in the studied area date back to that period, these substances can persist for more than a century [90,91]. Later, other activities contributed to the pollution. Since the establishment of glass factory, the whole processing has been powered by beech wood, coal, and coal for gas generators. Since 1961, mazut, fuel oil, butane, and propane were introduced, while natural gas began to be used in 1985 [92]. Moreover, mineral oils, used as lubricants in glass industry, were derived from the crude oil and almost exclusively contained PAHs [15,16]. Issues are also reflected in some raw materials that cannot be blended and in packaging waste, as stated in the guidelines [14].

Based on the data analysis, it is challenging to definitively determine whether the PTEs and PAHs share a common source or similar binding mechanism. If we consider the fact that the city of Paraćin with its surroundings is an important industry center, the impact cannot be ignored. Activities that have certainly contributed to PTEs emission and deposition in the study area over time are primarily the glass production, but also other industrial activities nearby as potential secondary sources of pollution: cement and construction material manufacturing, electrical and energy equipment production, and cable and metal hardware production. However, currently, it is impossible to determine individual influence or contribution share of secondary pollution sources due to the distance and climate conditions over the active period of these activities. These findings suggest the need for further investigation into physicochemical properties and pollutants, which is essential to accurately assess the soil condition and its suitability for future measures of remediation and use. To better determine these effects and establish monitoring, subsequent analyses are necessary. Such monitoring would include the determination of bioavailable forms of PTEs. Unlike many other industries, glass production is fundamentally designed with recycling in mind, especially in the container glass sector, where glass packaging is infinitely recyclable, making it a key element of circular economy strategies and environmental policy compliance under the Industrial Emissions Directive (IED). According to the latest statistics, 73% of all post-consumer glass packaging in the EU is collected for recycling without any degradation of its intrinsic properties if it is separately collected and treated.

In that regard, the assessment is also relevant from the regulatory perspectives. The EU Soil Strategy for 2030 and the proposed Soil Monitoring Law, among other objectives, aim to provide guidelines for soil restoration and reduce soil pollution to levels that are no longer harmful to people’s health or ecosystems. These documents aim to harmonize soil quality assessment across the EU and provide a framework that complements national regulations, such as Serbia’s Decree on Maximum Allowable Concentrations of Pollutants, Hazardous, and Dangerous Substances in Soil. To address the challenges regarding pollution sources, the Republic of Serbia has established a National Pollutant Release Register, a unique and structured database which contains data submitted by legal entities whose activities are identified as pollution sources. As of 2023, the Register includes 272 documented pollution sources across the country. It is maintained by the Environmental Protection Agency in accordance with the Law on Environmental Protection. In that regard, it provides a basis for risk assessment and strategic environmental management, with an aim to align with regional and international environmental regulations.

### 4.4. Limitations

Despite providing insight into historical and contemporary activities in the study area, as well as factors controlling the PTEs and PAH behavior, several limitations could be noted. These include the lack of historical emissions and soil data, limited depth coverage, overlapping industrial activities, uncertainties in precise pollution source determination, complex long-term processes which affect mobility and bioavailability, and the absence of direct evidence for historical PAH sources.

Additional limitations are related to health risk assessment. The gastrointestinal absorption parameter (GIABS) was not included due to the lack of site-specific toxicological data, while total Cr was used without speciation, which may affect the accuracy of carcinogenic risk assessment.

These aspects represent research gaps that could be addressed in future investigations.

## 5. Conclusions

This study analyzes the impact of past and present industrial activities on soil pollution within the glass factory complex. The most prominent contaminants in the studied area are PTEs, particularly zinc, nickel, copper, and cadmium, which exceeded both the MPC and RV in a considerable number of samples. Arsenic and mercury exceeded MPCs in a smaller number of samples. Around 32% of soil samples are moderately to highly polluted, with Pb, Cd, As, and Hg being the most prominent elements, contributing to the overall contamination (RI). The PAHs are unevenly present in soil, while LMW PAHs were more frequently detected, which indicates a predominantly pyrogenic origin of PAHs in industrial processes. Given the history of activities, it can be said that both PTEs and PAHs likely originate from multiple sources, including additives, fossil fuels, waste, etc. The dominance of different industrial activities indicates the complexity of the pollutants’ behavior in soil. Regarding influence on human health, the PTEs do not pose a non-carcinogenic risk (HI). In contrast, there is a high carcinogenic risk (TCR) associated with Ni, Cr, and As, with unacceptable levels in sporadic samples, with the ingestion being the primary exposure pathway to potential carcinogenic effects. These findings highlight the need for comprehensive and integrated environmental monitoring and prioritize the implementation of legal regulations and remediation measures. Considering historical and industrial legacy, monitoring represents ecological, human health, and cultural responsibility.

## Figures and Tables

**Table 1:** Basic statistical parameters of physical and chemical properties.

Parameter	Sand	Silt	Clay	pH	OC	CaCO_3_	Cl^−^	Fluorides
Unit	%	%	%		g kg^−1^	%	%	mg kg^−1^
N of samples	33	33	33	33	34	34	33	34
Min	32.20	1.10	1.90	7.28	10.71	0.00	0.0018	31.00
Max	97.00	32.30	40.10	8.62	164.48	43.96	0.0049	994.00
Mean	58.49	21.49	20.02	8.00	34.52	6.32	0.0029	292.23
SD	18.54	8.29	11.19	0.19	28.47	8.14	0.00077	175.53

SD—Standard deviation.

**Table 2:** Overview of PTEs concentrations (mg kg^−1^) and exceedance of regulatory values (Government of the Republic of Serbia, 2019).

	Zn	Cu	Pb	Ni	Cr	Cd	Hg	As	Sb	Fe
No. of samples in which PTEs were detected	34	34	34	34	34	31	4	34	5	34
Min	24.81	12.85	20.78	9.87	3.26	0.03	0.29	0.99	0.06	1054.35
Max	783.89	97.54	1111.19	150.62	62.18	6.71	0.44	79.90	2.07	65,657.90
Mean *	132.16	32.20	157.48	79.25	35.34	1.16	0.37	16.73	0.71	22,675.78
SD	123.00	14.85	252.46	33.47	12.24	1.35	0.06	13.78	0.92	11,019.26
IW value	160.97	1634.95	12.20	1296.90	493.33	0.00	0.00	33.71	37.33	>133,155.00
MPC	140	36	85	35	100	0.8	0.3	29	3	-
RV	720	190	530	210	380	12	10	55	15	-
No. > MPC	9	9	11	31	0	16	3	3	0	-
No. > RV	1	1	2	0	1	0	0	1	0	-
IW sample Exceeds MPC and/or RV	No	Yes	No	Yes	Yes	No	No	No	Yes	-

*—Mean value, excluding IW sample; SD—standard deviation; IW—sample of industrial waste; MPC—maximum permissible concentration (according to the regulation); RV—remediation value (according to the regulation).

**Table 3:** Overview of PAHs content (mg/kg) and exceedance of regulatory values for total PAHs.

PAH	Anthracene	Naphthalene	Phenanthrene	Benz[a] Anthracene	Benzo[k] Fluoranthene	Benzo[a] Pyrene	Benzo(g,h,i) Perylene	Chrysene	Fluoranthene	Indeno(1,2,3-cd) Pyrene	Total PAHs
No. of samples in which PAHs were detected	4	21	19	9	10	11	6	9	15	6	27
Min	0.042	0.033	0.032	0.046	0.032	0.032	0.048	0.043	0.037	0.047	0.036
Max	0.080	0.076	0.178	0.325	0.204	0.414	0.207	0.287	0.303	0.179	1.984
Mean	0.061	0.048	0.073	0.133	0.078	0.117	0.092	0.107	0.130	0.083	0.361
SD	0.027	0.012	0.046	0.092	0.054	0.115	0.058	0.076	0.093	0.049	0.464

SD—Standard deviation.

**Table 4:** Overview of the total non-carcinogenic risk (HI) and total carcinogenic risk (TCR).

	Zn	Cu	Pb	Ni	Cr	Cd	As	Sb	Hg
	HI								
min	7.1 × 10^−5^	2.8 × 10^−4^	1.8 × 10^−3^	9.5 × 10^−4^	1.1 × 10^−3^	9.9 × 10^−10^	1.9 × 10^−3^	0.0 × 10^0^	0.0 × 10^0^
max	2.3 × 10^−3^	3.5 × 10^−2^	1.6 × 10^−1^	1.2 × 10^−1^	1.7 × 10^−1^	6.6 × 10^−3^	1.6 × 10^−1^	9.6 × 10^−4^	1.1 × 10^−2^
mean	3.8 × 10^−4^	1.7 × 10^−3^	2.3 × 10^−2^	1.1 × 10^−2^	1.7 × 10^−2^	1.0 × 10^−3^	3.4 × 10^−2^	9.4 × 10^−5^	3.4 × 10^−4^
SD	3.5 × 10^−4^	5.9 × 10^−3^	3.7 × 10^−2^	2.0 × 10^−2^	2.7 × 10^−2^	1.3 × 10^−3^	2.7 × 10^−2^	2.6 × 10^−4^	1.8 × 10^−3^
	TCR								
min	N/A	N/A	8.9 × 10^−8^	7.1 × 10^−6^	1.5 × 10^−6^	5.2 × 10^−12^	1.5 × 10^−6^	N/A	N/A
max	N/A	N/A	8.1 × 10^−6^	9.3 × 10^−4^	2.3 × 10^−4^	3.5 × 10^−5^	1.2 × 10^−4^	N/A	N/A
mean	N/A	N/A	1.1 × 10^−6^	8.3 × 10^−5^	2.3 × 10^−5^	5.5 × 10^−6^	2.6 × 10^−5^	N/A	N/A
SD	N/A	N/A	1.8 × 10^−6^	1.5 × 10^−4^	3.8 × 10^−5^	6.9 × 10^−6^	2.1 × 10^−5^	N/A	N/A

SD Standard deviation; N/A not applicable (no carcinogenic slope factor available).

**Table 5:** Results of factor analysis (varimax rotation) for PTEs.

	Factor 1	Factor 2
Zn	0.498	0.413
Cu	**0.645**	0.338
Pb	**0.91**	−0.0447
Ni	0.444	**0.501**
Cr	0.0188	**0.899**
Cd	**0.553**	−0.0585
Fe	0.0391	**0.616**
As	**0.626**	0.366
Eigenvalue	3.01	1.24
Percent of Variance	59.632	24.62
Cumulative Percentage	59.632	84.252

Bold values indicate factor loadings greater than 0.5.

## Data Availability

The data that support the findings of this study may be available from the corresponding author upon reasonable request and with permission from the project funding organization.

## Supplementary Materials

The following supporting information can be downloaded at: https://www.mdpi.com/article/10.3390/toxics14040320/s1, Table S1. Toxicological parameters values for heavy metals; Table S2. Toxicological parameters for HI and TCR calculation; Table S3: Matrix correlation on some physicochemical soil properties, PTEs, and most common and total PAHs. References [30,32,53,93,94,95] in the manuscript are cited in the Supplementary Materials.

## Abbreviations

The following abbreviations are used in this manuscript:

PTE	Potentially toxic element
PAHs	Polycyclic aromatic hydrocarbons
IW	Industrial waste
MPC	Maximum permissible concentration
RV	Remediation Value
RI	Potential Ecological Risk Index
WRB	World Reference Base
SRPS	Serbian Standard
ISO	International Organization for Standardization
ASTM	American Society for Testing and Materials
AAS	Atomic absorption spectrometry
ICP-OES	Inductively coupled plasma–optical emission spectrometry
GC-MS	Gas chromatography–mass spectrometry
PI	Pollution Index
GB	Background concentration
Er	The Individual Ecological Risk Index
Cn	Concentration
Tr	Toxic response
TCR	Total carcinogenic risk index
ADD	Average daily doses
HI	Non-carcinogenic health risk index
FA	Factor analysis
OC	Organic carbon
SD	Standard deviation
LMW	Low-molecular weight
HMW	High-molecular weight
IED	Industrial Emissions Directive

## References

[1] Rodríguez Eugenio N., McLaughlin M.J., Pennock D.J. (2018). Soil Pollution: A Hidden Reality.

[2] Phiri R., Rangappa S.M., Siengchin S. (2024). Agro-waste for sustainable production: A review. J. Clean. Prod..

[3] Jia J., Bi C., Zhang J., Jin X., Chen Z. (2018). Characterization of PAHs in vegetables near industrial areas. Environ. Pollut..

[4] Zhang M. (2019). Challenges of soil and groundwater contamination. Synthesiology.

[5] Shetty S.S., Sonkusare S., Naik P.B., Kumari N.S., Madhyastha H. (2023). Environmental pollutants and human health. Heliyon.

[6] Gaur N., Sharma S., Yadav N. (2024). Environmental pollution. Green Chemistry Approaches to Environmental Sustainability.

[7] Casetta M., Philippe S., Courcot L., Dumoulin D., Billon G., Baudin F., Henry F., Hermoso M., Caillaud J. (2025). A quantitative assessment of the behavior of metallic elements in urban soils exposed to industrial dusts near Dunkerque (northern France). SOIL.

[8] Guan Y., Shao C., Gu Q., Ju M., Zhang Q. (2015). Method for assessing the integrated risk of soil pollution in industrial and mining gathering areas. Int. J. Environ. Res. Public Health.

[9] Kumar V. (2016). Impact of glass industry effluent disposal on soil characteristics. J. Environ. Health Sci..

[10] Yao C., Yang Y., Li C., Shen Z., Li J., Mei N., Luo C., Wang Y., Zhang C., Wang D. (2024). Heavy metal pollution in agricultural soils. Sci. Total Environ..

[11] Syms P. (2004). Previously Developed Land: Industrial Activities and Contamination.

[12] Manning D.A.C. (1995). Raw materials for the glass industry. Introduction to Industrial Minerals.

[13] Kuenen J., van der Most P., Rentz O., Nunge S., Trozzi C., Pulles T., Appelman W. (2013). EMEP/EEA Air Pollutant Emission Inventory Guidebook.

[14] European Bank for Reconstruction and Development (2014). Sub-Sectoral Environmental and Social Guideline: Glass Manufacturing.

[15] Menichini E., Bonanni L., Merli F. (1990). Determination of polycyclic aromatic hydrocarbons in mineral oils and oil aerosols in glass manufacturing. Toxicol. Environ. Chem..

[16] Tolbert P.E. (1997). Oils and cancer. Cancer Causes Control.

[17] Statista: Homepage. https://www.statista.com/homepage.

[18] FEVE Half Year 2022 Glass Packaging Production at Highest Levels. https://feve.org/half-year-2022-glass-packaging-production-at-highest-levels/.

[19] Pivić R., Stanojković Sebić A., Jošić D. (2013). Soil and plant contamination near highways. Pol. J. Environ. Stud..

[20] Zhang X., Lu W., Xu L., Wu W., Sun B., Fan W., Zheng H., Huang J. (2022). Environmental Risk Assessment of Polycyclic Aromatic Hydrocarbons in Farmland Soils near Highways: A Case Study of Guangzhou, China. Int. J. Environ. Res. Public Health.

[21] Bartkowiak A., Lemanowicz J., Rydlewska M., Sowiński P. (2024). The impact of proximity to road traffic on heavy metal accumulation and enzyme activity in urban soils and dandelion. Sustainability.

[22] Peng M., Yang Z., Liu Z., Han W., Wang Q., Liu F., Zhou Y., Ma H., Bai J., Cheng H. (2024). Heavy metals in roadside soils. Sci. Total Environ..

[23] Magiera T., Kyzioł-Komosińska J., Dzieniszewska A., Wawer M., Żogała B. (2020). Mobility of elements in historical glass-production wastes. Sci. Total Environ..

[24] Varun M., D’Souza R., Pratas J., Paul M.S. (2012). Metal contamination near glass industry. Environ. Sci. Pollut. Res..

[25] International Agency for Research on Cancer (1993). Exposures in the glass manufacturing industry. IARC Monographs on the Evaluation of Carcinogenic Risks to Humans.

[26] Irgolic K.J., Wacker W.E.C., Zingaro R.A. (1977). Chemistry of arsenic. Arsenic: Medical and Biological Effects of Environmental Pollutants.

[27] Newton R.G., Davison S. (1989). Conservation of Glass.

[28] Atkarskaya A.B., Bykov V.N. (2003). Clarification of glass using arsenic and antimony oxides. Glass Ceram..

[29] Mattisson K., Tekavec E., Lundh T., Stroh E. (2020). Metal exposure among children in glassworks areas. Int. J. Environ. Res. Public Health.

[30] Miletić A., Vesković J., Wang Y., Lučić M., Zhang Y., Onjia A. (2025). Occupational exposure to heavy metal(oid)-contaminated soil from mining operations: A case study of the Majdanpek Site, Serbia. Appl. Sci..

[31] Caković M., Beloica J., Belanović Simić S., Miljković P., Lukić S., Baumgertel A., Schwaiger F. (2021). Diffuse pollution and ecological risk assessment in Ludaš Lake Special Nature Reserve and Palić Nature Park. Forests.

[32] Belanović Simić S., Miljković P., Baumgertel A., Lukić S., Ljubičić J., Čakmak D. (2023). Environmental and health risk assessment due to potentially toxic elements in soil near former antimony mine in Western Serbia. Land.

[33] Mikić H. Serbian Glass Factory 1907–1945. https://creativeglassserbia.com/praeteritum/srpska-fabrika-stakla-1907-1945/.

[34] Mikić H. Serbian Glass Factory 1946–2013. https://creativeglassserbia.com/praeteritum/srpska-fabrika-stakla-1946-2013/.

[35] Marković Đ Serbian Glass Factory. https://sr.wikipedia.org/sr-ec/Датoтека:Српска_фабрика_стакла.jpg.

[36] Oktopaz Vaider Group/Paraćin Projects. https://www.oktopaz.com/sr/projects/.

[37] Aksić N., Pešić T., Filipović V., Todorović I. (2022). Srpska fabrika stakla u ogledalu vremena. Ethno-Cultural Annals for the Study of the Culture of Eastern Serbia and the Adjacent Areas: Enigma of the Serbian East–New Research Opportunities and Recapitulation.

[38] IUSS Working Group WRB (2022). World Reference Base for Soil Resources 2022. International Soil Classification System for Naming Soils and Creating Legends for Soil Maps.

[39] (2005). Soil Quality—Simplified Soil Description.

[40] (2007). Soil Quality—Determination of pH.

[41] (1987). Water for Analytical Laboratory Use—Specification and Test Methods.

[42] (2005). Soil Quality—Determination of Carbonate Content—Volumetric Method.

[43] (2005). Soil Quality—Determination of Total Nitrogen Content by Dry Combustion (Elemental Analysis).

[44] (2005). Soil Quality—Determination of Organic and Total Carbon after Dry Combustion (Elemental Analysis).

[45] (2019). Standard Test Methods for Chloride Ion in Water—Method B.

[46] (1995). Soil Quality—Extraction of Trace Elements Soluble in Aqua Regia.

[47] (2006). Soil Quality—Determination of Polycyclic Aromatic Hydrocarbons (PAH)—Gas Chromatographic Method with Mass Spectrometric Detection (GC-MS).

[48] Hakanson L. (1980). An ecological risk index for aquatic pollution control: A sedimentological approach. Water Res..

[49] Čakmak D., Pavlović P., Perović V., Mitrović M., Karadžić B., Jarić S., Pavlović M., Pavlović D., Mataruga Z., Marković M. (2018). Determination of Background Concentrations of Certain Harmful and Dangerous Substances in the Soil.

[50] Cheng H., Huang L., Ma P., Shi Y. (2019). Ecological risk and restoration measures relating to heavy metal pollution in industrial and mining wastelands. Int. J. Environ. Res. Public Health.

[51] Monaci F., Baroni D. (2025). Ecological risk of potentially toxic elements in peri-urban soils. Environ. Monit. Assess..

[52] USEPA (1989): Risk Assessment Guidance for Superfund, Volume I: Human Health Evaluation Manual (Part A), Interim Final, Office of Emergency and Remedial Response, EPA/540/1-89/0 02. https://www.epa.gov/sites/default/files/2015-09/documents/rags_a.pdf.

[53] Ying L., Shaogang L., Xiaoyang C. (2016). Assessment of heavy metal pollution and human health risk in urban soils of coal mining city in East China. Hum. Ecol. Rish Assess. Int. J..

[54] USEPA (2001). Risk Assessment Guidance for Superfund: Volume III-Part A, Process for Conducting Probabilistic Risk Assessment, 20460; Environmental Protection Agency, Office of Emergency and Remedial Response: Washington, DC, USA. EPA 540-R-02-002. https://www.epa.gov/sites/default/files/2015-09/documents/rags3adt_complete.pdf.

[55] Hu B., Shao S., Fu T., Fu Z., Zhou Y., Li Y., Lin Q., Chen S., Shi Z. (2020). Composite assessment of human health risk from potentially toxic elements through multiple exposure routes: A case study in farmland in an important industrial city in East China. J. Geochem. Explor..

[56] Reimann C., Filzmoser P., Garret R.G., Dutter R. (2008). Statistical Data Analysis Explained: Applied Environmental Statistics with R.

[57] Xu Z., Tsang D.C.W. (2022). Redox-induced transformation of toxic elements. Carbon Res..

[58] Piret N., Santoro R., Dogot L., Barthélemy B., Peyroux E., Proost J. (2018). Influence of glass composition on HF etching. J. Non-Cryst. Solids.

[59] Tanimoto S., Rehren T. (2008). Interactions between silicate and salt melts in glassmaking. J. Archaeol. Sci..

[60] Obranovic D., Barham D., Hancock R.G.V. (2002). Behaviour of chlorine in glass-melting. Proceedings of the 33rd International Symposium on Archaeometry.

[61] Government of the Republic of Serbia Uredba o Graničnim Vrednostima Zagađujućih, Štetnih i Opasnih Materija u Zemljištu. (eng. Regulation on Limit Values for Pollutants, Harmful and Hazardous Substances in Soil). https://www.paragraf.rs/propisi/uredba-granicnim-vrednostima-zagadjujucih-stetnih-opasnih-materija-zemljistu.html.

[62] Mrvić V., Zdravković M., Sikirić B., Čakmak D., Kostić-Kravljanac L. (2009). Content of harmful and dangerous elements. Plodnost i Sadržaj Opasnih i Štetnih Materija u Zemljištima Centralne Srbije.

[63] Šerbula S.M., Antonijević M.M., Milošević N.M., Milić S.M., Ilić A.A. (2010). Particulate matter and arsenic in Bor, Serbia. J. Hazard. Mater..

[64] Barać N., Škrivanj S., Bukumirić Z., Barać M., Manojlović D., Petrović R., Ćorac A. (2015). Arsenic in agricultural soils of a historically mined and industrial region of Southern Serbia and Northern Kosovo. Soil Sediment Contam..

[65] Lindsay W.L. (1972). Zinc in soils and plant nutrition. Adv. Agron..

[66] Kandakji T., Udeigwe T.K., Athanasiou D., Pappas S. (2015). Chemistry of arsenic in semi-arid alkaline soils. Water Air Soil Pollut..

[67] Brudzyńska P., Sionkowska A., Grisel M. (2023). Cotton textile dyeing by plant-derived colorants in the presence of natural additives. Fibers Polym..

[68] Smedley P.L., Kinniburgh D.G. (2002). Arsenic in natural waters: A review. Appl. Geochem..

[69] Aide M., Beighley D., Dunn D. (2016). Arsenic in the soil environment: A soil chemistry review. Int. J. Appl. Agric. Res..

[70] Rotter P., Kuta J., Vácha R., Sáňka M. (2017). Role of Mn and Fe oxides in soil retention. Plant Soil Environ..

[71] Saljnikov E., Mrvić V., Čakmak D., Jaramaz D., Perović V., Antić-Mladenović S., Pavlović P. (2019). Heavy metal pollution near thermal power plant. Environ. Geochem. Health.

[72] Augustsson A., Åström M., Bergbäck B., Elert M., Höglund L.O., Kleja D.B. (2016). High metal reactivity and environmental risks at a site contaminated by glass waste. Chemosphere.

[73] Turner A. (2018). Migratable lead and cadmium on drinking glassware. Sci. Total Environ..

[74] Thalassinos G., Petropoulos S.A., Grammenou A., Antoniadis V. (2023). Potentially toxic elements in soils. Agriculture.

[75] Pavlović D., Pavlović M., Perović V., Mataruga Z., Čakmak D., Mitrović M., Pavlović P. (2021). Risk assessment of toxic elements in urban soils. Int. J. Environ. Res. Public Health.

[76] Alloway B.J. (2013). Heavy Metals in Soils.

[77] Aljumaily M.M., Al-Hamandi H., Al-Obaidi M.A., Al-Zidan R.R. (2022). The effect of calcium carbonate content on the zinc quantity–intensity relationship in some soils of Mosul, Iraq. Cienc. Tecnol. Agropecu..

[78] Owabor C.N., Oboh I.O., Omiojieahior F.A. (2011). Adsorption isotherms for naphthalene. Adv. Mater. Res..

[79] Osagie E.I., Owabor C.N. (2015). Adsorption of naphthalene on soils. Adv. Chem. Eng. Sci..

[80] Jia N., Li L., Guo H., Xie M. (2024). Important role of Fe oxides in global soil carbon stabilization. Nat. Commun..

[81] Ukalska-Jaruga A., Smreczak B. (2020). Impact of organic matter on PAHs in soils. Molecules.

[82] Ye C., Huang W., Hall S.J., Hu S. (2022). Organic carbon and iron oxides at global scale. Glob. Biogeochem. Cycles.

[83] Schvartz C., Denaix L., Douay F., Perdrix E., Sterckeman T., Wroblewski A., Charbonnier P., Ledesert B. (1999). Pathways of Pb, Cd and Zn transfer. Modelling of Transport Processes in Soils.

[84] Michels F., Ribeiro J., Henriques M.H. (2025). Soil contamination by industries in a protected area. Environ. Geochem. Health.

[85] Kabata-Pendias A. (2011). Trace Elements in Soil and Plants.

[86] Charvalas G., Molla A., Lolas A., Skoufogianni E., Papadopoulos S., Chatzikirou E., Emmanouil C., Christopoulou O. (2025). Evaluation of potential toxic elements in soils from three urban areas surrounding a steel industrial zone. Toxics.

[87] Abraham J., Dowling K., Florentine S. (2017). Risk of post-fire metal mobilization into surface water resources: A review. Sci. Total Environ..

[88] Bowd E.J., Banks S.C., Strong C.L., Lindenmayer D.B. (2019). Long-term impacts of wildfire and logging on forest soils. Nat. Geosci..

[89] Golia E.E., Kantzou O.D., Chartodiplomenou M.-A., Papadimou S.G., Tsiropoulos N.G. (2023). Study of potentially toxic metal adsorption in polluted acid and alkaline soils. Soil Syst..

[90] Lundstedt S. (2003). Analysis of PAHs and Their Transformation Products in Contaminated Soil and Remedial Processes. Ph.D. Thesis.

[91] Froger C., Saby N.P.A., Jolivet C.C., Boulonne L., Caria G., Freulon X., de Fouquet C., Roussel H., Marot F., Bispo A. (2021). Spatial variations, origins, and risk assessments of polycyclic aromatic hydrocarbons in French soils. SOIL.

[92] Mikić H. Kreativno Staklo Srbije: Paraćinsko Staklarstvo–Tehnologije i Tehnike. https://creativeglassserbia.com/.

[93] USEPA (2024). Regional Screening Level (RSL) Composite Worker Soil Table (TR = 1E-06, HQ = 1). https://www.epa.gov/risk/regional-screening-levels-rsls-generic-tables.

[94] Antoniadis V., Shaheen S.M., Levizou E., Shahid M., Niazi N.K., Vithanage M., Ok Y.S., Bolan N., Rinklebe J. (2019). A critical prospective analysis of the potential toxicity of trace element regulation limits in soils worldwide: Are they protective concerning health risk assessment?—A review. Environ. Int..

[95] Isinkaye O.M. (2018). Distribution and multivariate pollution risks assessment of heavy metals and natural radionuclides around abandoned iron-ore mines in North Central Nigeria. Earth Syst. Environ..

