# Supplementary material for: Industrial Legacy and Glassmaking: Ecological and Human Health Risk Assessment in Paraćin, Serbia
Source: Toxics. 2026 Apr 12;14(4):320. doi: 10.3390/toxics14040320 (PMC13120367; doi:10.3390/toxics14040320)
Supplement: Supplementary file 1 [file toxics-14-00320-s001.zip › toxics-4228530-supplementary.pdf]

Supplement of

Industrial Legacy and Glassmaking: Ecological and Human Health Risk Assessment in Paraćin, Serbia

Predrag Miljković, Jelena Beloica, Snežana Belanović Simić, Stefan Miletić
University of Belgrade – Faculty of Forestry, Department of Ecological Engineering for Soil and Water Resources Protection, 11030 Belgrade, Serbia
Correspondence to: Predrag Miljković (predrag.miljkovic@sfb.bg.ac.rs)

Table S1. Toxicological parameters values for heavy metals

	**Reference dose**			**Dermal absorption factor**	**Cancer slope factor**			**Inhalation unit risk**
	**RfDing**	**RfDinh**	**RfDdermal**	**ABS**	**CSFing**	**CSFinh**	**CSFdermal**	**IUR**
	**mg $kg^{-1}day^{-1}$**			**unitless**	**(mg $kg^{-1}day^{-1})^{-1}$**			**(μg $m^{-3})^{-1}$**
Zn	3.00E-01	3.00E-01	6.00E-02	0.001	-	-	-	-
Cu	4.00E-02	4.02E-02	1.20E-02	0.001	-	-	-	-
Pb	3.50E-03	3.52E-03	5.30E-04	0.001	8.50E-03	4.20E-02	-	1.20E-05
Ni	2.00E-02	2.06E-02	5.40E-03	0.001	8.40E-01	-	-	2.60E-04
Cr	3.00E-03	3.00E-05	6.00E-05	0.001	5.00E-01	4.20E+01	2.00E+01	8.40E-02
Cd	1.00E-03	1.00E-05	1.00E-05	0.001	6.10E+00	6.30E+00	6.10E+00	1.80E-03
As	3.00E-04	3.00E-04	1.23E-04	0.03	1.50E+00	1.51E+01	3.66E+00	4.30E-03
Sb	4.00E-04	-	-	0.001	-	-	-	-
Hg	3.00E-03	9.00E-05	2.00E-05	0.001	-	-	-	-
Reference	[1, 2]	[2]	[2]	[3]	[2, 4]	[2]	[2]	[1, 3]

Table S2. Toxicological parameters for *HI* and *TCR* calculation

**Parameter**	**Unit**	**Value**	**Reference**
CS	mg kg^{-1}	measured	-
IngR	mg day^{-1}	100	[2, 5]
InhR	m^3 day^{-1}	20	[5]
EF	Days/year	250	[2]
ED	Years	25	[2]
BW	kg	80	[2]
AT	Days	365 x ED	[4]
SA	cm^2	6032	[4]
SL	mg cm^{-2} day^{-1}	0.07	[6]
PEF	m^3 kg^{-1}	1.36E+09	[6]

Table S3: Matrix correlation on some physico-chemical soil properties, PTEs, most common and total PAHs

	pH	$CaCO_3$	OC	Zn	Cu	Pb	Ni	Cr	Cd	Fe	As	Sand	Silt	Clay	Fluorides	Cl^-	benzo[a]anthracene	benzo[a]anthracene	benzo[a]anthracene	benzo[a]anthracene	benzo[a]anthracene	Naphthalene	Phenanthrene	Total PAHs
pH	1																							
$CaCO_3$	0.56***(33)	1																						
OC	-0.35*(33)	0.09(34)	1																					
Zn	0.54**(33)	0.77***(34)	0.22(34)	1																				
Cu	-0.17(33)	-0.13(34)	0.03(34)	0.06(34)	1																			
Pb	-0.09(33)	0.02(34)	0.22(34)	0.07(34)	-0.07(34)	1																		
Ni	0.01(33)	-0.14(34)	0.01(34)	0.05(34)	0.99***(34)	-0.02(34)	1																	
Cr	-0.24(33)	-0.20(34)	-0.01(34)	0.00(34)	0.99***(34)	-0.09(34)	0.99***(34)	1																
Cd	-0.03(31)	0.25(31)	0.22(31)	0.16(31)	0.26(31)	0.49**(31)	0.22(31)	-0.04(31)	1															
Fe	-0.75***(33)	-0.38*(33)	0.42*(33)	-0.23(33)	0.26(33)	-0.11(33)	0.01(33)	0.42*(33)	-0.02(31)	1														
As	-0.64***(33)	-0.22(34)	0.58***(34)	-0.14(34)	0.23(34)	0.18(34)	0.23(34)	0.24(34)	0.49**(31)	0.75***(33)	1													
Sand	0.15(33)	0.27(33)	0.22(33)	0.10(33)	0.05(33)	0.21(33)	0.09(33)	-0.30(33)	0.18(31)	-0.38*(33)	0.16(33)	1												
Silt	-0.14(33)	-0.12(33)	-0.16(33)	0.03(33)	0.00(33)	-0.09(33)	-0.01(33)	0.26(33)	-0.09(31)	0.37*(33)	-0.08(33)	-0.93***(33)	1											
Clay	-0.16(33)	-0.35*(33)	-0.25(33)	-0.19(33)	-0.08(33)	-0.28(33)	-0.15(33)	0.30(33)	-0.24(31)	0.36*(33)	-0.21(33)	-0.96***(33)	0.81***(33)	1										
Fluorides	-0.20(33)	0.02(34)	0.07(34)	0.09(34)	-0.25(34)	0.56***(34)	-0.20(34)	-0.22(34)	0.26(31)	0.18(33)	0.19(34)	-0.08(33)	0.22(33)	-0.03(33)	1									
Cl^-	0.09(33)	0.19(33)	0.20(33)	0.11(33)	0.13(33)	0.05(33)	-0.04(33)	-0.21(33)	0.18(31)	-0.22(33)	-0.03(33)	0.41*(33)	-0.38*(33)	-0.40*(33)	-0.18(33)	1								
Benzo[a]anthracene	-0.90***(9)	-0.41(9)	0.80*(9)	0.24(9)	0.57(9)	0.33(9)	-0.17(9)	0.09(9)	0.49(8)	0.90**(9)	0.87**(9)	0.39(9)	-0.30(9)	-0.43(9)	0.28(9)	0.12(9)	1							
Benzo[k]fluoranthene	-0.86**(10)	-0.22(10)	0.73*(10)	-0.07(10)	0.45(10)	0.09(10)	-0.20(10)	-0.03(10)	0.77*(9)	0.89***(10)	0.89***(10)	0.44(10)	-0.35(10)	-0.45(10)	0.44(10)	0.36(10)	0.91***(9)	1						
Benzo[a]pyrene	-0.93***(11)	-0.16(11)	0.76**(11)	-0.09(11)	0.48(11)	0.11(11)	-0.10(11)	-0.05(11)	0.34(10)	0.90***(11)	0.94***(11)	0.33(11)	-0.26(11)	-0.35(11)	0.48(11)	0.02(11)	0.88**(9)	0.92***(10)	1					
Chrysene	-0.93***(9)	-0.21(9)	0.35(9)	-0.12(9)	0.05(9)	-0.02(9)	-0.43(9)	-0.12(9)	-0.17(8)	0.92***(9)	0.85**(9)	0.25(9)	-0.04(9)	-0.40(9)	0.60(9)	-0.18(9)	0.79*(9)	0.68*(9)	0.74*(9)	1				
Fluoranthene	-0.56*(15)	0.10(15)	0.65(15)	0.25(15)	0.62*(15)	0.49(15)	-0.19(15)	-0.17(15)	0.31(14)	0.56*(15)	0.47(15)	0.19(15)	-0.16(15)	-0.19(15)	0.01(15)	0.40(15)	0.75*(9)	0.63(10)	0.59(10)	0.54(9)	1			
Naphthalene	0.37(21)	0.47*(21)	0.01(21)	0.46*(21)	-0.06(21)	0.10(21)	-0.26(21)	-0.55**(21)	0.33(19)	-0.33(21)	-0.10(21)	0.35(21)	-0.34(21)	-0.34(21)	-0.15(21)	0.53*(21)	0.40(7)	0.57(8)	0.24(8)	0.13(7)	0.15(11)	1		
Phenanthrene	-0.01(19)	0.31(19)	0.51*(19)	0.27(19)	0.51*(19)	0.73***(19)	0.07(19)	0.05(19)	0.65**(18)	0.13(19)	0.24(19)	0.00(19)	-0.04(19)	0.04(19)	-0.08(19)	0.14(19)	0.26(8)	0.04(8)	0.08(8)	-0.11(8)	0.73(12)	0.10(17)	1	
Total PAHs	-0.59**(27)	-0.10(27)	0.70***(27)	-0.11(27)	0.39*(27)	0.07(27)	-0.08(27)	0.05(27)	0.04(25)	0.68***(27)	0.79***(27)	0.17(27)	-0.12(27)	-0.19(27)	0.07(27)	0.19(27)	0.97(9)	0.91(10)	0.91(11)	0.82(9)	0.88(15)	-0.08(21)	0.43(19)	1

Notes: Values represent Pearson correlation coefficients. Significance levels: * $p < 0.05$, ** $p < 0.01$, *** $p < 0.001$. Numbers in parentheses indicate sample size (n).

References

1. USEPA, Regional Screening Level (RSL) Composite Worker Soil Table (TR=1E-06, HQ=1) November 2024 https://www.epa.gov/risk/regional-screening-levels-rsls-generic-tables (accessed 22 February 2026)
2. Mliletić, A., Vesković, J., Wang, Y., Huang, X., Lučić, M., Zhang, Y., Onjia, A. (2025). Occupational Exposure to Heavy Metal(loid)-Contaminated Soil from Mining Operations: A Case Study of the Majdanpek Site, Serbia, Applied Sciences 15(19), 10711; https://doi.org/10.3390/app151910711
3. Antoniadis, V., Shaheen, S.M., Levizou, E., Shahid, M., Niazi, N.K., Vithanage, M., Ok, Y.S., Bolan, N., Rinklebe, J. (2019). A critical prospective analysis of the potential toxicity of trace element regulation limits in soils worldwide: Are they protective concerning health risk assessment? - A review, Environment International, Volume 127, June 2019, Pages 819-847, https://doi.org/10.1016/j.envint.2019.03.039
4. Belanović Simić, S., Miljković, P., Baumgertel, A., Lukić, S., Ljubičić, J., Čakmak, D. (2022). Environmental and Health Risk Assessment Due to Potentially Toxic Elements in Soil near Former Antimony Mine in Western Serbia, *Land*, *12*(2), 421; **https://doi.org/10.3390/land12020421**
5. Ying, L.; Shaogang, L.; Xiaoyang, C. Assessment of heavy metal pollution and human health risk in urban soils of coal mining city in East China. *Human and ecological risk assessment: An international journal*. **2016,** 22(6), https://doi.org/10.1080/10807039.2016.1174924
6. Isinkaye, O. M. Distribution and multivariate pollution risks assessment of heavy metals and natural radionuclides around abandoned iron-ore mines in North Central Nigeria. *Earth Syst. Environ.* **2018,** *2*, 331–343. https://doi.org/10.1007/s41748-018-0035-0
